# Coupled Hybrid Continuum-Discrete Model of Tumor Angiogenesis and Growth

**DOI:** 10.1371/journal.pone.0163173

**Published:** 2016-10-04

**Authors:** Jie Lyu, Jinfeng Cao, Peiming Zhang, Yang Liu, Hongtao Cheng

**Affiliations:** 1 Medical Instrumentation School, Shanghai University of Medicine and Health Sciences, Shanghai, 201318, China; 2 Periodicals Agency, Shanghai University, Shanghai, 200444, China; Universita degli Studi di Bari Aldo Moro, ITALY

## Abstract

The processes governing tumor growth and angiogenesis are codependent. To study the relationship between them, we proposed a coupled hybrid continuum-discrete model. In this model, tumor cells, their microenvironment (extracellular matrixes, matrix-degrading enzymes, and tumor angiogenic factors), and their network of blood vessels, described by a series of discrete points, were considered. The results of numerical simulation reveal the process of tumor growth and the change in microenvironment from avascular to vascular stage, indicating that the network of blood vessels develops gradually as the tumor grows. Our findings also reveal that a tumor is divided into three regions: necrotic, semi-necrotic, and well-vascularized. The results agree well with the previous relevant studies and physiological facts, and this model represents a platform for further investigations of tumor therapy.

## Introduction

Tumors, especially malignant tumors, are one of the most important causes of death in humans. In recent decades, tumor growth has received increasing attention from researchers. The emergence of new technologies in molecular physiology has led to a rapid development of research on the physiological processes governing tumor growth. Mathematical oncology, a branch of tumor research, has also gained gradual attention. Together with mathematical modeling and numerical simulation, mathematical oncology explores the theoretical basis of the pathological and physiological phenomena, simulates the process, and predicts the trend of tumor growth. This can ultimately provide new ideas and approaches towards clinical research on tumors.

The process regulating tumor growth is very complex, including cell-cell interactions, interactions between tumor cells and extracellular matrices (ECM), and proliferation and death of tumor cells. Besides, the concentrations of chemicals in the microenvironment can affect tumor growth. Among the mathematical models of tumor growth, the hybrid mathematical model, which combines continuous and discrete models, is widely used. Its continuous-model component describes the changing process of chemicals in the tumor microenvironment, based on the reaction-diffusion equation; while its discrete cellular-model component describes the cell movements and interactions, based on the principle of random walk. The hybrid model can simulate the tumor growth more effectively. Anderson et al. [1] presented two types of mathematical models, which describe the invasion of host tissue by tumor cells. The models focus on three key variables implicated in the invasion process, namely, tumor cells, ECMs, and matrix-degrading enzymes (MDEs). Later, Anderson[2] improved the model by considering the effects of cell adhesion. Subsequent series-hybrid models of tumor growth were proposed based on Anderson's model[3–6]. Recently, Zhou et al.[7] presented a multiscale continuum-discrete model to simulate avascular tumor growth, based on the effects of p27 gene. Additionally, Lyu et al.[8] built a hybrid model of tumor growth considering static capillary points.

Incipient tumor is in the avascular stage, while after a certain period, the tumor shifts towards the vascular stage and displays a characteristic formation of microvascular network. In 1971, Folkman[9] first put forward the hypothesis of tumor angiogenesis. He believed that tumor growth depends on angiogenesis. In the angiogenesis stage, tumor cells first secrete a series of chemicals called tumor angiogenic factors (TAFs). The TAFs spread, resulting in a concentration difference between the tumor and the blood vessels nearby. The concentration difference can lead the proteolytic enzymes to degrade the matrix surrounding the blood vessels. Subsequently, the endothelial cells of the parent vessel accumulate to form capillary sprouts, which move towards the tumor and form the microvasculature.The angiogenesis provides the tumor with oxygen, nutrients, growth factors, etc., making it the source of tumor growth. In terms of mathematical models, Anderson and Chaplain[10] took diffusion, chemotaxis, and haptotaxis of endothelial cells into account, built a two-dimensional discrete model for angiogenesis, tracked the trajectories of endothelial cells by judging the movement direction of endothelial cells, described the process of tumor microvasculature, and obtained a visual vascular network. Subsequently, Chaplain[11] extended Anderson and Chaplain's model into a three-dimensional space. Wu et al.[12] presented a three-dimensional model of solid tumor angiogenesis including arteriole, capillary, and venule, and studied the blood perfusion in intravascular and interstitial spaces in tumor microvasculature. This model is now used for development of antiangiogenic treatments [13–15].

**Fig 1:**
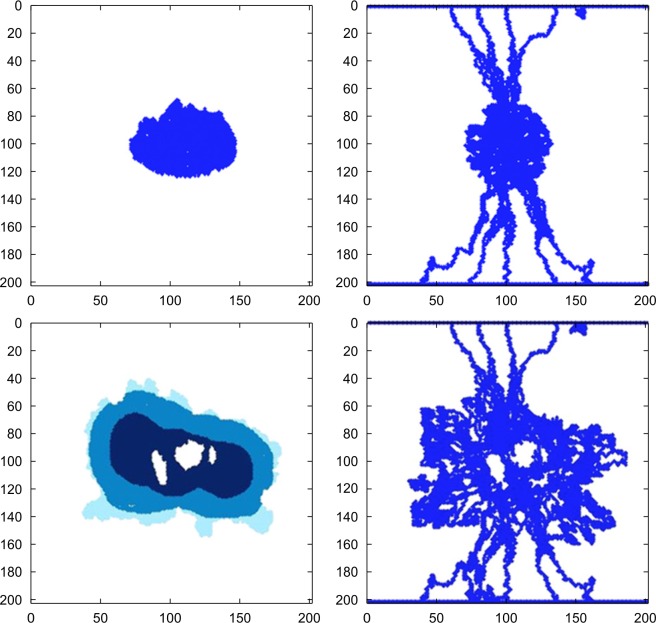
Distribution of tumor cells (left) and blood vessel networks (right)[16].

In fact, tumor growth and angiogenesis are dependent processes. Angiogenesis affects tumor growth, while tumor cells affect angiogenesis by changing the tumor microenvironment. Therefore, the coupled model of angiogenesis and tumor growth has received more and more attention. Cai et al.[16; 17] performed interesting research in this area. They built a coupled mathematical model of angiogenesis, tumor growth, and blood perfusion, analyzed the hemodynamics, and gradually modified the initial network of blood vessels, using wall shear stress (WSS) as the basis for vascular collapse. In their model, the change in tumor microenvironment simultaneously affects tumor growth. However, to calculate hemodynamics by Cai et al.’s model, an initial blood vessel network must be set first, and the blood vessels are modified instead of being generated. Fig 1 shows the initial distribution of tumor cells and blood vessel networks in the first row, and shows the simulation results after 24 days in the second row.

In mathematical modeling, blood vessel networks are commonly represented as pipe networks[18]. Pipe networks are closer to real tumor microvascular networks, which have been widely used in tumor blood dynamics. However, in the numerical simulation of tumor angiogenesis, endothelial cell division, migration, and proliferation should be taken into account. Meanwhile, network connectivity should be tested, and corrections should be made during blood dynamics calculations. The complex rules lead to a large amount of calculations. If coupled with the process of tumor growth, it will further increase the amount of calculations and reduce the efficiency of the numerical simulation. Therefore, we believe that using point networks is an effective way to describe the network of blood vessels. First, the material transport in the tumor area is mainly due to a diffusion effect, while the convection effect has little influence. Therefore, it is reasonable to ignore the convection effect and use point networks instead of blood vessels. Second, the oxygen distribution is associated with the density of blood vessels. In the model, we can adjust the blood vessel points to change the density of blood vessels. Consequently, we only need to establish a proper model to control blood points’generation, which is relatively simple. Here, we propose a two-dimensional coupled mathematical model of tumor growth and angiogenesis, using the hybrid modeling method. In the model, a series of discrete points are used to describe the blood vessel network. The process governing tumor growth and the change in microenvironment from avascular to vascular stage can be described through numerical simulation. The model has the advantage of including only small amounts of calculations, is suitable for numerical simulation, and takes into account both radiation and chemical therapies.

## Mathematical model

In the previous work, four important variables of tumor growth have been considered, including the density of tumor cells *n*(*x*,*t*), the concentration of ECM *f*(*x*,*t*), the concentration of MDE *m*(*x*,*t*) and the concentration of oxygen *c*(*x*,*t*). The complete system of equations describing the interactions between these factors is as follows[2]:

$$\frac{\partial n}{\partial t}=\overset{Random\;motility}{\overbrace{D_{n}\nabla^{2}n}}-\overset{Haptotaxis}{\overbrace{\chi \nabla ∙(n\nabla f)}}$$ (1)

$$\frac{\partial f}{\partial t}=-\overset{Degradation}{\overbrace{\delta mf}}$$ (2)

$$\frac{\partial m}{\partial t}=\overset{Diffusion}{\overbrace{D_{m}\nabla^{2}m}}+\overset{Production}{\overbrace{\mu n}}-\overset{Decay}{\overbrace{\lambda m}}$$ (3)

$$\frac{\partial c}{\partial t}=\overset{Diffusion}{\overbrace{D_{c}\nabla^{2}c}}-\overset{Uptake}{\overbrace{\gamma n}}-\overset{Decay}{\overbrace{\alpha c}}$$ (4)

In Eq 1, *D*_*n*_ is the diffusion coefficient of tumor cells, and *χ* is the haptotaxis coefficient of tumor cells. In Eq 2, *δ* is the degradation coefficient of ECMs. In Eq 3, *D*_*m*_ is the diffusion coefficient of MDEs, *μ* is the generation rate of MDEs, and *λ* is the decay rate of MDEs. In Eq 4, *D*_*c*_ is the diffusion coefficient of oxygen, γ is the consumption rate of oxygen by tumor cells, and *α* is the natural decay rate. In this paper, the concentration of TAFs *a*(*x*,*t*) as a new variable is added to the mathematical model. The equation is as follows,

$$\frac{\partial a}{\partial t}=\overset{Diffusion}{\overbrace{D_{a}\nabla^{2}a}}+\overset{Production}{\overbrace{\varepsilon n}}-\overset{Decay}{\overbrace{\theta a}}$$ (5)

TAFs are assumed to be transported by pure diffusion. They are produced by tumor cells, and consumed naturally at a certain rate. In Eq 5, *D*_*a*_ is the diffusion coefficient of TAFs, *ε* is the generation rate of TAFs and *θ* is the natural decay rate.

We consider Eqs 1–5 as non-dimensional. Let the length scale be *L* = 1 cm(the tumor radius in the avascular phase is usually 1–2mm and the radius continues to increase in the angiogenesis stage) and the time scale be *τ* = 16 h(the average time taken for mitosis to occur is approximately 8–24h). The appropriate reference variables of the tumor cell density, ECM concentration, MDE concentration, oxygen concentration, and TAF concentration are denoted by *n*^0^, *f*^0^, *m*^0^, *c*^0^, and *a*^0^, respectively. Therefore, setting

$$\tilde{n}=\frac{n}{n^{0}},\tilde{f}=\frac{f}{f^{0}},\tilde{m}=\frac{m}{m^{0}},\tilde{c}=\frac{c}{c^{0}},\tilde{a}=\frac{a}{a^{0}},\tilde{x}=\frac{x}{L},\tilde{t}=\frac{t}{\tau }$$

and substituting them into Eqs 1–5, we can obtain the scaled system of equations with superscripts omitted,

$$\frac{\partial n}{\partial t}=\overset{Random\;motility}{\overbrace{d_{n}\nabla^{2}n}}-\overset{Haptotaxis}{\overbrace{\rho \nabla ∙(n\nabla f)}}$$ (6)

$$\frac{\partial f}{\partial t}=-\overset{Degradation}{\overbrace{\eta mf}}$$ (7)

$$\frac{\partial m}{\partial t}=\overset{Diffusion}{\overbrace{d_{m}\nabla^{2}m}}+\overset{Production}{\overbrace{\kappa n}}-\overset{Decay}{\overbrace{\sigma m}}$$ (8)

$$\frac{\partial c}{\partial t}=\overset{Diffusion}{\overbrace{d_{c}\nabla^{2}c}}-\overset{Uptake}{\overbrace{\omega n}}-\overset{Decay}{\overbrace{\phi c}}$$ (9)

$$\frac{\partial a}{\partial t}=\overset{Diffusion}{\overbrace{d_{a}\nabla^{2}a}}+\overset{Production}{\overbrace{\beta n}}-\overset{Decay}{\overbrace{\psi a}}$$ (10)

where

$$d_{n}=\frac{\tau D_{n}}{L^{2}},\rho =\frac{\tau \chi f^{0}}{L^{2}},\eta =\tau m^{0}\delta ,\;d_{m}=\frac{\tau D_{m}}{L^{2}},\kappa =\frac{\tau \mu n_{0}}{m^{0}},\sigma =\tau \lambda ,$$

$$d_{c}=\frac{\tau D_{c}}{L^{2}},\omega =\frac{\tau n^{0}\gamma }{c^{0}},\phi =\tau \alpha ,d_{a}=\frac{\tau D_{a}}{L^{2}},\beta =\frac{\tau \varepsilon n^{0}}{a^{0}},\psi =\tau \theta ,$$

in which all variables are positive. The phenomenon of tumor cells producing TAFs is related to the oxygen concentration. Therefore, we assume

$$\beta =\left\{ \begin{array}{l} \;0,\;c>0.4\\ \beta_{std},\;c\leq 0.4 \end{array}\right.$$ (11)

which indicates that tumor cells are under anoxic conditions, and secrete TAFs when *c* ≤ 0.4, otherwise, tumor cells do not secrete TAFs. The values of *ρ*, *ω*, and *κ* are associated with the oxygen concentration and predominate a linear relationship, that is *ρ* = (3*c* + 0.7)*ρ*_std_, *ω* = (3*c* + 0.7)*ω*_std_, and *κ* = (3*c* + 0.7)*κ*_std_

TAFs cannot form blood vessels directly. However, they can induce endothelial cells to migrate to a region with high TAF concentration and activate endothelial cells to turn into blood vessels. The vascular network that this study focuses on is the distribution of blood points rather than continuous blood vessels. This study does not establish the motion equation of endothelial cells, and does not consider the motion process of endothelial cells or the process of blood vessels gradually developing from parent vessels to the tumor area. Instead, this study uses a probability model and simulates the blood vessels in the tumor area by generating blood points. Assume a random position *A*, and the probability of generation of vascular point in position *A* is

$$P_{b}=\left\{ \begin{array}{l} \;p_{c},\;\frac{r}{r_{0}}\leq 0.5\\ pa,\;\frac{r}{r_{0}}>0.5 \end{array}\right.$$ (12)

where *r*_0_ denotes the radius of tumor, and *r* denotes the distance between *A* and the center of tumor. Under the influence of chemotaxis, endothelial cells move towards a region where the TAF concentration is high. However, with increase in the TAF concentration, the sensitivity of endothelial cells to chemotaxis decreases[19]. In the center of the tumor, the oxygen is less and the TAF concentration is higher. This study assumes that the area of $\frac{r}{r_{0}}\leq 0.5$ has a low possibility of endothelial cells entering, therefore, the probability of generation of vascular point(*p*_*c*_) is set to 0. Outside the area, the probability of generation of vascular points is proportional to the TAF concentration, where *p* is the proportional coefficient. When the vascular point is generated, it will operate as a source of oxygen for the surrounding area, by diffusion. Consequently, the number of vascular points and their respective positions affect the oxygen distribution pattern within the tumor region, thereby affecting tumor growth. At the cellular level, the processes of an individual tumor cell's migration are taken into account, including proliferation, mutation, death, and intercellular adhesion. The details are available in [8].

The simulation area is 1cm ×1cm, uniformly divided into 201× 201 lattices. The space step is Δ*x* = Δ*y* = 0.005. The time step is Δ*t* = 0.002. No-flux boundary conditions are imposed on the grids, which means that the tumor cells, ECM, MDE, and oxygen are restricted within the domain. The initial condition is as follows: 81 tumor cells are localized around the center, with a random age ranging from 0 to 16h, the ECM concentration on each grid point is a random value between 0 and 1, the MDE concentration is 0 throughout the domain, the oxygen concentration is set to 1, and there is no vascular point in the domain. The simulation runs for 35,000 steps, which is equivalent to 1120h.

The non-dimensional parameters are[2; 11]

$$d_{n}=5\times 10^{-6},d_{m}=5\times 10^{-6},d_{c}=5\times 10^{-4}(outside\;of\;the\;tumor),$$

$$d_{c}=2.5\times 10^{-4}(inside\;of\;the\;tumor),d_{a}=5\times 10^{-6},\rho_{std}=10^{-4},\eta =50,$$

$$\kappa_{std}=1,\sigma =0,\omega_{std}=0.6,\phi =0.05,\beta_{std}=0.025,\psi =0.01,p=1.$$

**Fig 2:**
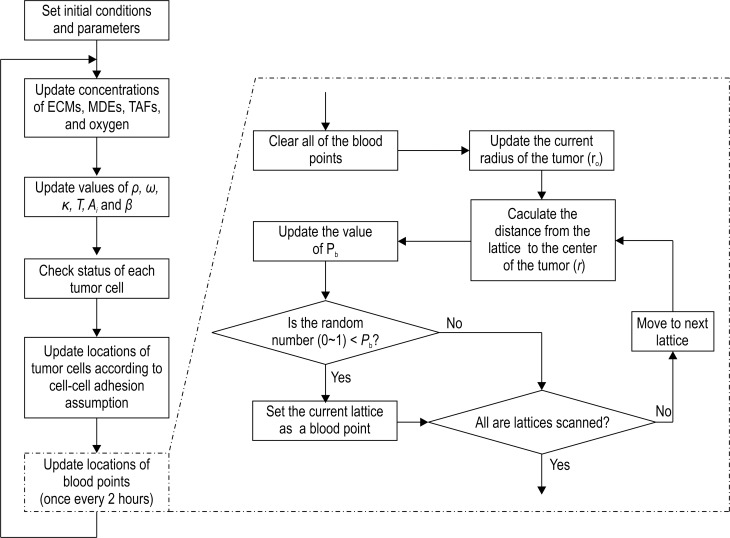
Simulation process of the mathematical model.

The simulation process is shown in Fig 2.

In Fig 2, the oxygen concentration maintains the value of 1 in vascular points. The distribution of vascular points is updated every 2 h. This is because the capillaries inside the tumor are often seen as a temporary component. With the development of the tumor, the capillaries are likely to be influenced by the microenvironment, which leads them to change, refract, or even die.

## Simulation results

### The process of tumor growth and change in microenvironment

**Fig 3:**
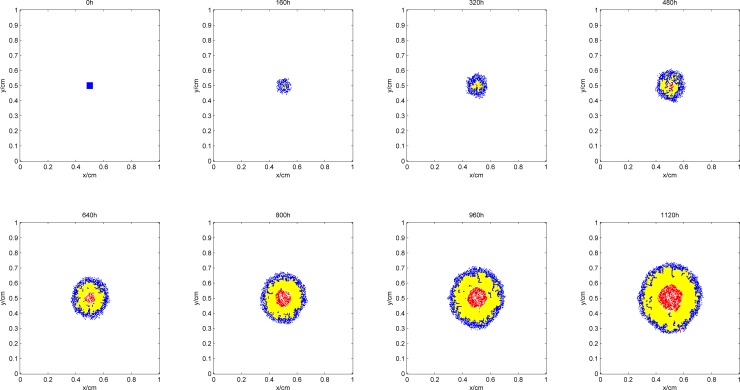
Process of tumor growth during the simulation.

**Fig 4:**
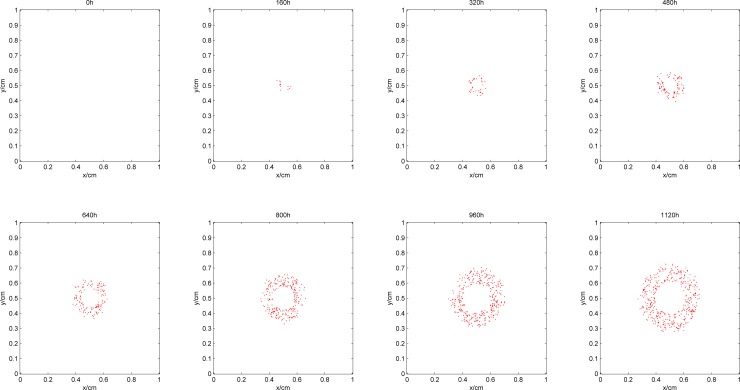
Development of blood vessel network during the simulation.

**Fig 5:**
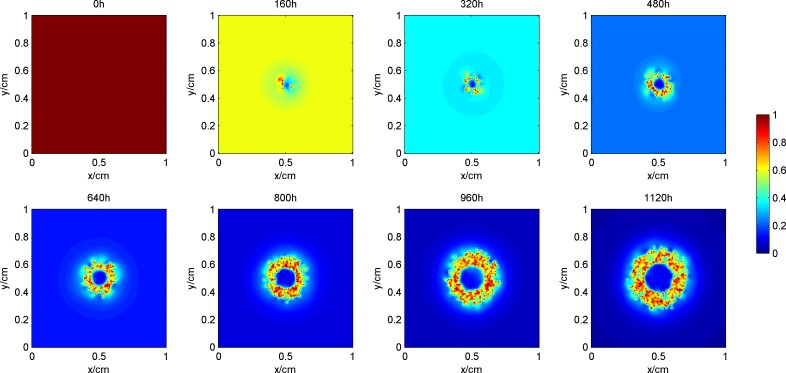
Distribution of the oxygen concentration during the simulation.

**Fig 6:**
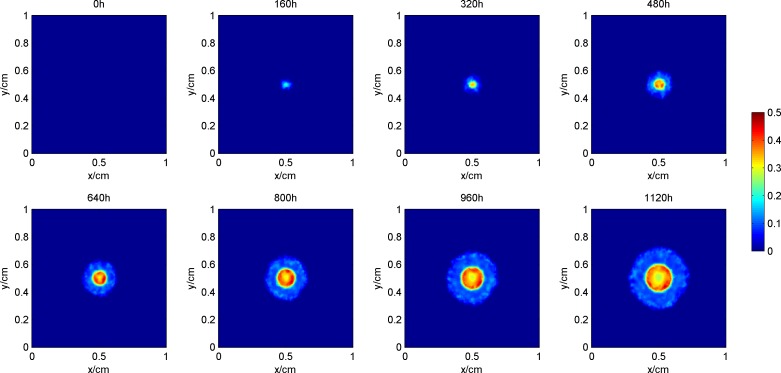
Distribution of the TAF concentration during the simulation.

Fig 3 shows the process of tumor growth during the simulation, where the blue area denotes proliferative cells, yellow denotes quiescent cells, and red denotes dead cells. Fig 4 shows the synchronization of the vascular network as the tumor grows. Figs 5 and 6 give the distribution of the oxygen concentration and the TAF concentration at each moment, respectively.

In the early phase of tumor growth, the oxygen is sufficient within the simulation area, the number of tumor cells is small, and there is enough room for tumor proliferation. Therefore, the proliferative cells are more abundant than others are. After 320h, as the radius of the tumor increases, the activity space in the center of the tumor is restricted. Although the oxygen concentration shows a certain degree of decline, it can still maintain tumor cell survival. The proliferative cells begin to turn into quiescent cells within this area. Meanwhile, hypoxic cells begin to secrete TAF, inducing blood vessel points around the tumor. Since the TAF concentration in the simulation area at this pointis low and the distribution of the region is also limited, blood vessel points only appear in the center of the tumor to improve its oxygen environment. From 480h, with further increase in the radius of the tumor, the cells located in the center of the tumor cannot get enough oxygen. The oxygen concentration is lower than 0.1, which means that the tumor cells are in an extreme oxygen deprivation situation and start to die. As the hypoxia situation gets worse, the TAF concentration increases, the distribution range expands, and the number of blood vessel points becomes larger. Subsequently, the radius of the tumor and the number of blood vessel points increase at the same time. However, the form of the tumor remains unchanged, that is, the necrosis sits in the center, proliferative cells are in the outer layer, and a large number of quiescent cells are in the middle layer. In the center of the tumor, due to the lack of blood vessels, oxygen in the external area can hardly get inside by diffusion. Therefore, the oxygen concentration in the area is lower than 0.1. However, in the middle and outer layers of the tumor, with intensive blood vessels, the hypoxia situation can be improved. The distribution of TAF concentrations is opposite to that of oxygen. The TAF concentration is higher in the center of the tumor, while in the middle and outer layers it is remarkably reduced because the blood vessel points improve the hypoxia condition.

**Fig 7:**
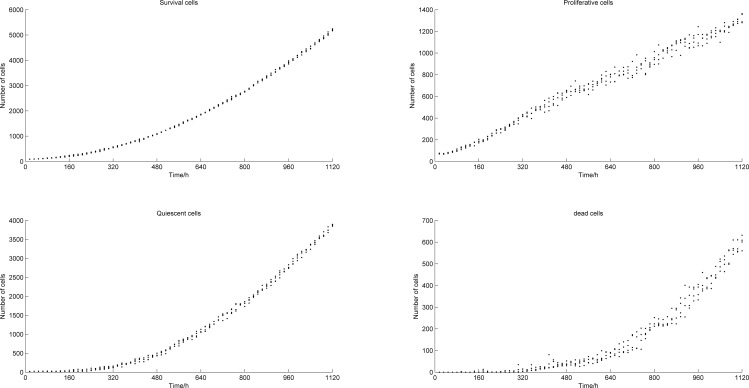
Numbers of tumor cells vs. time (4 simulations).

**Fig 8:**
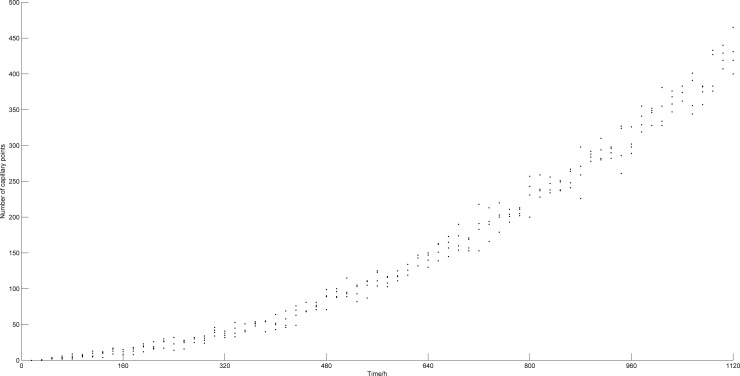
Numbers of blood vessel points vs. time (4 simulations).

The generation of blood points and tumor growth mainly follow random patterns. In order to examine the impact of random factors on the results, and test the repeatability of the model, we repeated the numerical simulation four times without changing the model parameters. Figs 7 and 8 show the increase in number of tumor cells and blood vessels with time, respectively. Here, the four simulation results are shown in discrete points. It can be observed that, even when random factors are included in the model, the trends that the numbers of tumor cells and blood points follow along time are still consistent.

Table 1 shows the average numbers and standard deviations of tumor cells and blood points at 1120 h, for all 4 simulations. From this table, we can see that the random factors have less effect on the final simulation results, and therefore, that the model yields good repeatability.

**Table 1:** Numbers of tumor cells and blood points at 1120 h.

	Numbers
Survival cells	5201.25 ± 35.63
Proliferative cells	1322.75 ± 43.81
Quiescent cells	3878.5 ± 21.30
Dead cells	600.5 ± 30.03
Capillary points	430.25 ± 27.66

### The change in numbers of tumor cells and blood points

**Fig 9:**
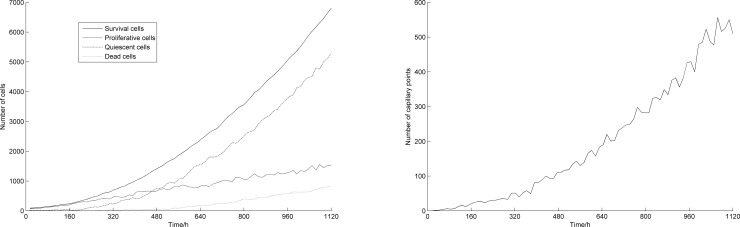
Numbers of tumor cells and blood vessel points vs. time.

**Fig 10:**
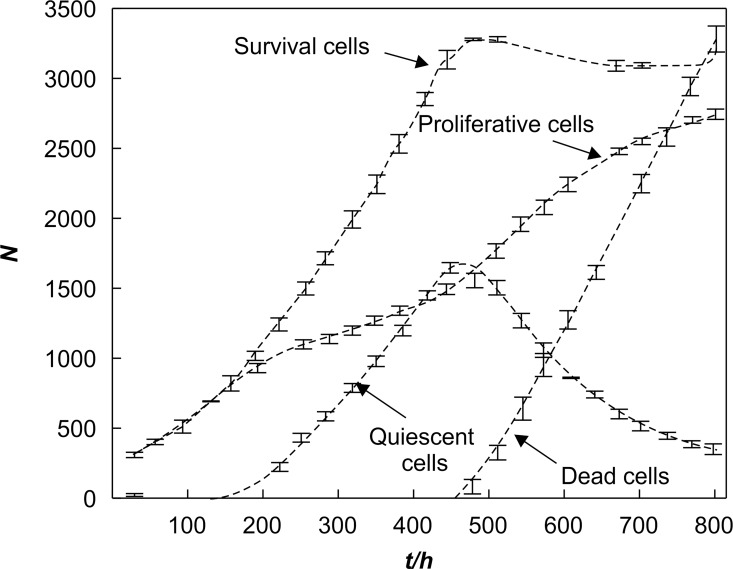
Numbers of tumor cells vs. time (without considering angiogenesis) [8]. The distribution of tumor cells and blood points.

Fig 9 shows the numbers of tumor cells and blood vessel points vs. time, respectively. Fig 10 describes the relationship between the number of tumor cells and time without taking into account angiogenesis [8], which generates significant differences with Fig 9. From Fig 10, we observe that after 450 h, the number of tumor cells tends to stabilize. This happens because when no angiogenesis occurs, the density of blood vessels in the microenvironment of the tumor stays constant and the oxygen is limited. Tumor cells cannot grow indefinitely. In this study, we show that the vascular network is gradually formed after introducing angiogenesis into the model and as the tumor grows. The oxygen supply is significantly improved so that the number of tumor cells can increase further, which causes the tumor to develop from the avascular stage to the vascular stage.

Usually, when considering the distribution of its inner vascular network, the tumor can be divided into three regions. The outer region has numerous blood vessels, while the middle region is semi-necrotic, with fewer blood vessels than the outer region. The inner region is a necrotic region with no vascular distribution.

**Fig 11:**
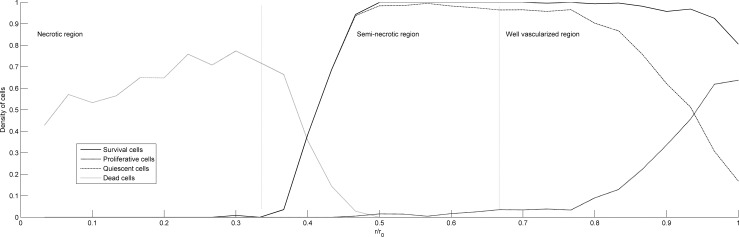
Distribution of the tumor cells density along the radial direction of the tumor.

Fig 11 shows the distribution of the tumor cells density along the radial direction of the tumor obtained by numerical simulation. It is observed that the necrotic region mostly contains dead cells. In the semi-necrotic region, the density of dead cells begins to fall sharply, and the density of survival cells increases greatly. The quiescent cells represent a large portion of the survival cells, which indicates that, in the semi-necrotic region, the oxygen concentration is sufficient to ensure tumor cells survival, even though there is not enough space for them to migrate. In the well-vascularized region, the density of live cells remains high. However, their composition changes drastically. The density of quiescent cells begins to decrease sharply. Instead, the density of proliferative cells rises. This indicates that the space available has largely increased. The distribution of tumor cells is consistent with the clinical observation.

**Fig 12:**
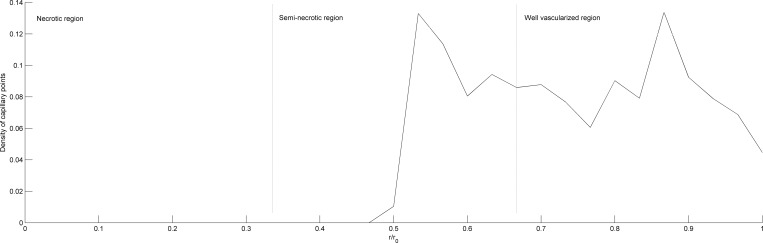
Distribution of the density of blood vessels along the radial direction of the tumor.

Fig 12 presents the distribution of the density of blood vessels along the radial direction of the tumor. In the necrotic region, the density of blood vessels is 0. In the semi-necrotic region, the blood vessel points begin to appear around $\frac{r}{r_{0}}=0.5$ and the density increases greatly. In the well-vascularized region, the density remains at 9%, and has a peak reaching about 13%. Near the edge of the tumor, the density shows an obvious drop. The results are in good agreement with those obtained by Wu et al. using a 3D7P model[19].

## Sensitivity analysis of parameters in point probability function

In the presented coupled model, the probability function of vascular point generation (Eq 12) is key. It determines the distribution of blood points in the process of tumor growth, affects the tumor microenvironment, and as a result, influences tumor growth. Above, we made some hypotheses regarding the parameters. Now, we will discuss these hypotheses.

### (1) Effect of *P*_*c*_

**Fig 13:**
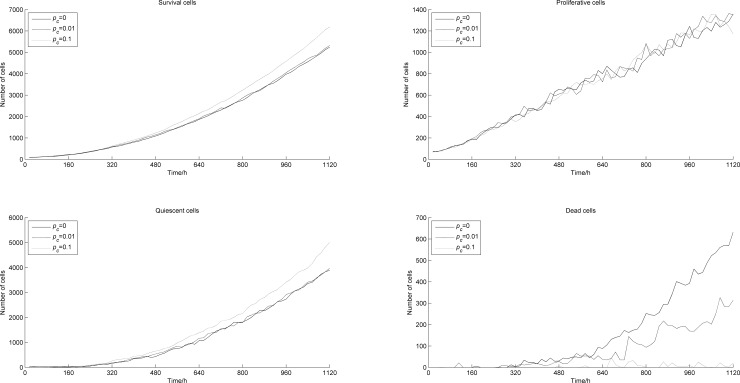
Numbers of tumor cells vs. time with different *p*_*c*._

**Fig 14:**
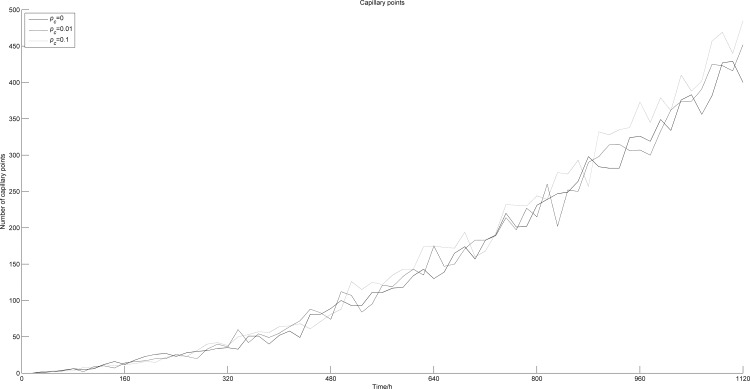
Numbers of blood vessel points vs. time with different *p*_*c*._

*P*_*c*_ denotes the probability of blood points in the center of the tumor. Fig 13 shows the change sin tumor cell numbers along time with different *P*_*c*._ When *P*_*c*_ = 0.1, the number of survival cells increases significantly, and this increase almost only concerns quiescent cells. In contrast, the number of dead cells is sharply reduced, almost down to zero. This is because when *P*_*c*_ = 0.1, a small amount of blood points appears in the center of the tumor, improving the anoxic condition and helping the tumor cells in this area to survive. Since the increasing survival cells locate in the center of the tumor, and there is no proliferation space, the cells turn into quiescent cells. We also noticed that the number of proliferative cells did not change with *P*_*c*_. This is because proliferative cells are mainly located at the edge of the tumor, where the generation of blood points is not affected by *P*_*c*_, and the microenvironment almost remains the same. Fig 14 shows the number of blood points appearing along time with different *P*_*c*_. When *P*_*c*_ increases, the number of blood points increases accordingly, but at a small rate.

### (2) Effect of *p*

**Fig 15:**
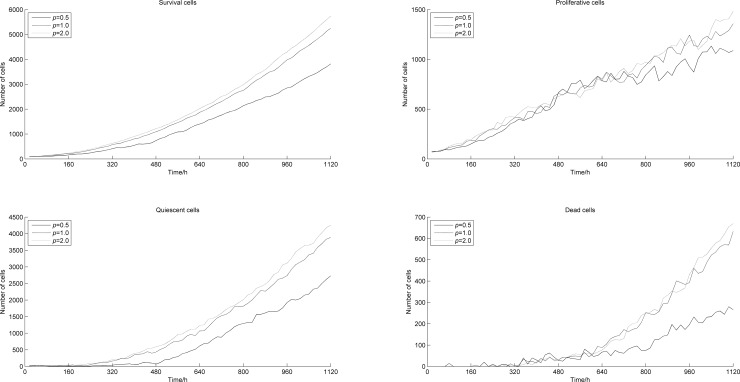
Numbers of tumor cells vs. time with different *p*.

**Fig 16:**
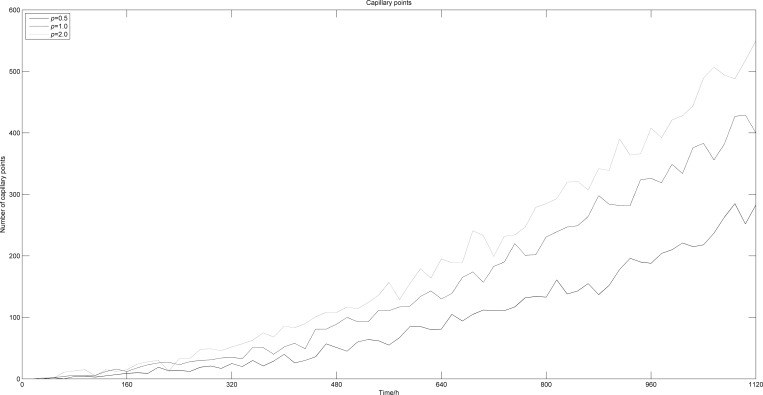
Numbers of blood vessel points vs. time with different *p*.

Under the same TAF concentration, the probability of blood points generation increases with *p*. Fig 15 shows the number of tumor cells appearing along time with different *p*. With the increase of *p*, the number of survival and dead cells both increases, which means that the tumor volume increases considerably. The increase in numbers of cells at the tumor’s edge leads to an increment of the oxygen concentration, which makes tumor cells more likely to easily move around and expand. In this case, the number of proliferative cells increases, leading to an accelerated tumor growth. Besides, when comparing two examples (*p* = 1 and *p* = 2) from Figs 15 and 16, we notice that the difference in numbers of blood points is greater than the fluctuation observed in numbers of tumor cells. This suggests that when *p* = 1, the vascular points have generated an oxygen concentration close to saturation at the edge of the tumor. A further increase of *p* can generate more blood points, with a more extended distribution, while having a limited impact on the increase in oxygen concentration, and therefore, little influence on tumor growth.

## Conclusions

Based on previous works, this study introduces the concept of TAFs and puts forward the probability model of blood vessel points of the tumor, using the distribution of TAF concentrations. This study also shows that the shift in tumor microenvironment from avascular to vascular phase is related to the increase in the blood vessel density points from 0 upwards; and establishes a new, coupled model of tumor growth and angiogenesis. Through numerical simulation, this model can be used to visualize changes in the tumor microenvironment, growth of blood vessels, distribution of oxygen concentrations, and TAF concentrations during tumor growth.

Angiogenesis of tumor is a complicated process. Researchers have found that the reaction of tumor cells to lack of oxygen and acidic environments involves unclear mechanisms of gene expression modulation. Tumor cells induce and promote the TAFs, which activate endothelial cells lined up on the wall of the host blood vessel. Subsequently, the endothelial cells migrate towards the tumor by chemotaxis.

Eventually, the vascular network of the tumor is formed. In this study, we used a probability model to generate blood vessel points, without considering the motion and modifications of endothelial cells. Therefore, in an attempt to validate the credibility of this model, we found that both the location of tumor cells and blood vessel points, or the changes in the numbers of tumor cells and blood vessel points, are in agreement with relevant previous results as well as established physiological facts of tumor growth. Therefore, this model is reliable and can be used as a platform for further investigations on tumor therapy.

## Supporting Information

S1 File MATLAB program of the model. (M)
